# Spinal Anesthesia in the Prone Position: A Case Series

**DOI:** 10.7759/cureus.108801

**Published:** 2026-05-13

**Authors:** Beatriz Xavier, Cristina P Sousa, Susana Maia, Alexandra Carneiro, José Cardoso

**Affiliations:** 1 Anesthesiology, Unidade Local de Saúde de Trás-os-Montes e Alto Douro, Vila Real, PRT; 2 Anesthesiology, Centro Hospitalar de Trás-os-Montes e Alto Douro, Vila Real, PRT; 3 Anesthesiology, Hospital da Luz-Setúbal, Setúbal, PRT

**Keywords:** cerebrospinal fluid, neuraxial anesthesia, pilonidal sinus, prone position, spinal anaesthesia

## Abstract

Background

Spinal anesthesia (SA) is a safe and effective strategy for many procedures, providing several advantages over general anesthesia. SA is usually performed in a sitting or lateral position, followed by careful repositioning to achieve the final surgical position. This mobilization process may cause technical difficulties related to the patient, consume valuable time, and lead to an unpredictable spread of the local anesthetic agent. To overcome these challenges, some authors have successfully performed SA with the patient already in the prone position.

Methods

In this article, we present a case series of 81 patients who underwent pilonidal sinus surgery under SA in the prone position.

Results

The technique achieved a 100% overall success rate, with 86.4% success on the first attempt. Adequate anesthetic block was obtained in all cases, without hemodynamic instability or respiratory complications during the procedure, and no complications related to the technique or patient positioning were observed.

Conclusions

SA performed in the prone position appears to be a safe and effective approach for pilonidal sinus surgery, providing high success rates and a low incidence of complications.

## Introduction

Spinal anesthesia (SA) is a safe and effective alternative to general anesthesia for a variety of procedures. It provides several advantages, such as reduced blood loss, fewer perioperative cardiac ischemic events, lower incidence of postoperative hypoxia, reduced risk of arterial and venous thrombosis, enhanced postoperative pain management, shorter recovery time, lower analgesic requirements, and higher patient satisfaction rates [1-3].

This technique is usually performed with the patient in either a sitting or lateral position, followed by careful repositioning to achieve optimal surgical access. This mobilization can be particularly challenging when transitioning them to a prone position, especially in obese patients. In addition to the imposed difficulty, it is time-consuming and repositioning could potentially lead to an unpredictable spread of SA, causing hemodynamic instability [3].

To overcome this issue, some authors have successfully performed SA with the patient in the prone position [4,5]. This approach allows patients to place themselves comfortably for surgery, eliminating the need for additional mobilization. This reduces the risk of pressure injuries, lightens the workload on medical staff, and helps prevent hemodynamic changes, ensuring more consistent drug distribution and block level. Additionally, performing SA in the prone position with a low dose of a hypobaric solution could allow for more targeted sensory nerve root blockade while minimizing motor root involvement, achieving a more selective block.

However, managing patients in the prone position presents some challenges, including ensuring proper needle insertion, accounting for the baricity of the local anesthetic, considering the effects of gravity on cerebrospinal fluid (CSF) flow and drug distribution, monitoring hemodynamic changes, and maintaining patient comfort throughout the perioperative period. In addition to these challenges, the limited experience in performing SA in the prone position presents an added difficulty. In this article, we present a case series where surgery for pilonidal sinus excision was performed under SA in patients already in the prone position. This study aimed to evaluate the feasibility and safety of SA performed in the prone position for pilonidal sinus surgery, while also describing the technical aspects of the procedure and the associated perioperative clinical outcomes.

## Materials and methods

This retrospective case series was conducted at Hospital da Luz Setúbal, including all patients who underwent pilonidal sinus surgery under SA in the prone position between January and April 2025. Inclusion criteria comprised adult patients (≥18 years) scheduled for elective pilonidal sinus surgery using this anesthetic technique. Patients with contraindications to SA, refusal to undergo the procedure, known allergy to local anesthetics, or incomplete medical records were excluded.

We retrieved the medical records of 81 patients who underwent pilonidal sinus excision under SA performed in the prone position, from January to April of 2025. Demographic and clinical data were collected from electronic medical records, including age, sex, anthropometric parameters, procedural details, and perioperative outcomes.

Since data were used for presentation as a case series, ethnic committee approval was unnecessary according to local policy. Following pre-anesthetic evaluation, SA under light to moderate sedation was offered to the patient as the primary anesthetic plan, and informed consent was obtained.

**Figure 1:**
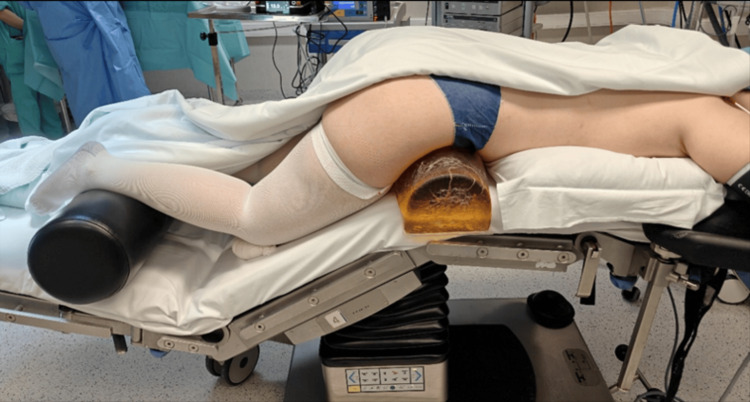
Patient positioning in the prone position for the performance of a subarachnoid block

The patients were placed in the prone position with a gel roll under the pelvis and pillows under the chest and abdomen, knees semi-flexed, thighs flexed to 60°, and arms abducted at the shoulders and flexed at the elbows, as can be seen in Figure 1.

The goal was to facilitate lumbar spine flexion and increase interlaminar space. Additional padding was provided on pressure points. All patients were monitored according to the American Society of Anesthesiologists (ASA) standards. Patients were sedated with ketamine 10 mg and propofol 20-30 mg before performing the SA.

All blocks were performed under aseptic technique at L4-L5 or L5-S1, depending on the assessment of the anesthesiologist, to which level seemed best in each patient. A 27 G 80 mm Sprotte needle with an introducer was used in all but one morbidly obese patient (body mass index 47.8 kg/m^2^), who required a 25 G 120 mm Quincke needle (27 G 120 mm was unavailable at the time). Due to the prone positioning, it was usually necessary to do a gentle aspiration to ensure CSF reflux through the needle and confirm it was in the subarachnoid space. After that, the operating table was placed in a 20° Trendelenburg position, and hypobaric 0.5% levobupivacaine (Fresenius Kabi, Bad Homburg, Germany) 4.5-6 mg and sufentanil 2 mcg were administered. Local anesthetic dosing was individualized according to patient height, whereby shorter patients received lower doses to achieve an appropriate sensory block.

After five minutes, the effectiveness of the saddle block for the surgical procedure was assessed. Adequacy of the anesthetic block was verified through the absence of pain to pinching stimuli applied over the sacral area, achieving a block at least at the L5-S1 level. The patients remained in the same position, without the need for repositioning during the entire surgery. A propofol 10-20 mg bolus was administered throughout the procedure to titrate light to moderate sedation for patient comfort.

Multimodal analgesia consisted of intravenous acetaminophen 1 g and ketorolac 30 mg. Ondansetron 4 mg was given for nausea and vomit prophylaxis. At the end of surgery, patients were able to return to their own beds independently. Due to operating room management, all patients had their surgical procedures in the late evening and remained at the hospital overnight, being discharged on the morning after the procedure.

## Results

Eighty-one patients were included. Their demographic characteristics are summarized in Table 1.

**Table 1:** Demographic characteristics of the patients Continuous variables presented as mean ± 2 SD. SD: standard deviation

Variable	Population (n = 81)
Sex (male:female)	68 (84.0%):13 (6.0%)
Age (years)	24.6 ± 15.6
Height (cm)	173.7 ± 16.4
Weight (kg)	78.3 ± 31.7
Body mass index (kg/cm^2^)	25.7 ± 9.1
<25 kg/m^2^	43 (53.1%)
25-29.9 kg/m^2^	28 (34.6%)
30-34.9 kg/m^2^	7 (8.6%)
35-39.9 kg/m^2^	2 (2.5%)
≥40 kg/m^2^	1 (1.2%)

The anesthetic technique was successfully performed in all patients without complications. In most patients, 70 (86.4%), the SA was done at the first attempt, 9/81 (11.1%) patients required two attempts, and 2/81 (2.5%) patients required three attempts. The level attempted was chosen at the sole discretion of the anesthesiologist. The average duration of the surgery was approximately 20 minutes. The patients remained hemodynamically stable, spontaneously breathing, and comfortable throughout the whole procedure. Key procedural and outcome data are summarized in Table 2.

**Table 2:** Key procedural and outcome data

Variable	Population (n = 81)
Spinal interspace used	
L4-L5	57 (70.4%)
L5-S1	24 (29.6%)
Dose of levobupivacaine	
4.5 mg	2 (2.5%)
5 mg	11 (13.6%)
5.5 mg	48 (59.3%)
6 mg	20 (24.7%)
Use of sufentanil	81 (100%)
Number of attempts	
1	70 (86.4%)
2	9 (11.1%)
3	2 (2.5%)
Complications	
Vasopressor requirement	0
Assistance with ventilation	0
Need for anesthetic supplementation	0
Total procedural time	30-45 min

## Discussion

SA is typically performed in the sitting or lateral position. The failure rate of SA ranges from 1% to 17%, with variability in success rates potentially attributed to differences in the population and the anesthesiologist’s experience [6]. Experienced practitioners report the failure rate to be less than 1% when performing SA in the conventional position [7].

In this work, the authors present a case series demonstrating the safe and effective use of SA in the prone position. The series includes 81 patients who successfully underwent pilonidal surgery with this approach. The block was performed by an experienced anesthesiologist, without failed blocks documented, which is consistent with the reported success rate.

This approach to SA offers advantages over the standard approach. A clear advantage is the reduction of the risk of positioning-related injuries, since the patients placed themselves comfortably, mitigating the risk of injuries from overstretching the peripheral nerves beyond normally tolerated limits or excessive joint extension [8]. Additionally, puncturing in the prone position with low doses of hypobaric solution primarily targets the sensory nerve roots, while minimizing the blockade of motor roots. This is achieved due to the anatomical arrangement of spinal nerves, where the posterior roots constitute the sensory nerves and the anterior roots function as the motor nerves [4]. This selective blockade may represent an advantage not only for anorectal surgery, as in the cases presented, but it can also be extrapolated to orthopedic or spinal surgery, where preserving motor strength is beneficial [9,10]. The authors achieved a selective sensory block in all patients, without motor block, as all patients were able to move independently to their beds at the end of the procedure.

Finally, the need to mobilize the patient after SA can be challenging, especially in cases of obesity, as nearly half of our population had at least some degree of excess weight. In frail or trauma patients, repositioning them after SA can increase the risk of added injuries. In addition to this imposed difficulty, it is time-consuming, and mobilizing them could potentially lead to an unpredictable spread of SA, causing hemodynamic instability [3].

Despite the numerous advantages of administering SA in the prone position, it presents technical challenges for anesthesiologists, and there is a learning curve. In this case series, the technique was successfully performed on the first try in 70 patients, achieving a first attempt success rate of 86.4%. For the remaining patients, two attempts were required in nine cases (11.1%) and three attempts in two cases (2.5%). There were no cases when the technique had to be abandoned.

Commonly encountered issues include having a good interlaminar space opening, hence the importance of placing a pillow under the abdomen to correct lumbar lordosis and enhance interlaminar space, and confirming needle placement in the subarachnoid space. Observing CSF reflux after reaching the intrathecal space is not always easy due to gravity. To overcome this challenge, gentle aspiration of CSF is recommended when getting a pop feeling after dural puncture to ensure correct needle placement. In these cases, as the anesthesiologist used their right hand (dominant hand), they positioned themselves on the patient's left side. Despite the challenges associated with this technique, the high first-attempt success rate reported by the authors (86.4%) was attributed to the careful positioning of the patient, as previously described and illustrated in Figure 1, as well as to the requirement for CSF aspiration to confirm correct placement within the subarachnoid space.

This study has several limitations that should be acknowledged. First, its single-center design may limit the generalizability of the findings to other settings with different clinical practices and levels of expertise. Second, the relatively modest sample size may have reduced the statistical power to detect smaller differences in clinical outcomes. Third, the absence of a control group precludes direct comparison with other techniques or patient positions. Finally, as an operator-dependent procedure, the performance of the SA in the prone position may not be reproducible in less experienced hands.

## Conclusions

SA in the prone position remains an underutilized technique despite many procedures being done in patients in the prone position. However, this approach could offer an optimal surgical exposure, eliminating the need for additional mobilization before the surgery and thus minimizing hemodynamic and respiratory instability associated with it. In all 81 patients, SA was performed with a success rate of 100%, 86.4% in the first attempt, and without complications. While challenges may arise during this technique, prone SA proved to be a viable, efficient, and safe technique for pilonidal sinus surgery, avoiding the need for intraoperative repositioning and consistently providing stable conditions.
